# Pivotal Functions of Plasmacytoid Dendritic Cells in Systemic Autoimmune Pathogenesis

**DOI:** 10.4172/2155-9899.1000212

**Published:** 2014-04-22

**Authors:** Wei Cao

**Affiliations:** Department of Immunology, The University of Texas MD Anderson Cancer Center, Houston, Texas, USA

**Keywords:** Type I interferon, Systemic lupus erythematosus, Autoimmune disease, Plasmacytoid dendritic cells, Toll-like receptor, Amyloid, Nucleic acid, Innate immune activation, Lupus model, Immune complex

## Abstract

Plasmacytoid dendritic cells (pDCs) were initially identified as the prominent natural type I interferon-producing cells during viral infection. Over the past decade, the aberrant production of interferon α/β by pDCs in response to self-derived molecular entities has been critically implicated in the pathogenesis of systemic lupus erythematosus and recognized as a general feature underlying other autoimmune diseases. On top of imperative studies on human pDCs, the functional involvement and mechanism by which the pDC-interferon α/β pathway facilitates the progression of autoimmunity have been unraveled recently from investigations with several experimental lupus models. This article reviews correlating information obtained from human *in vitro* characterization and murine *in vivo* studies and highlights the fundamental and multifaceted contribution of pDCs to the pathogenesis of systemic autoimmune manifestation.

## Introduction

Since the discovery of the first mammalian toll-like receptor (TLR) nearly 20 years ago [1,2], the importance of the innate immune response in shaping the outcome of adaptive immune reactions has become better appreciated. The well-orchestrated functions of sensing, activation, and feedback from the innate immune system are crucial to the elicitation of a proper adaptive immune response to protect the host against invasion by microbial agents or expansion of neoplasm and to maintain an immunological balance to avoid unnecessary self-damage. The pathogenesis of autoimmune diseases has long been studied primarily from the perspective of abnormalities in the adaptive immune system, which are important effectors of disease manifestation. It is now understood that aberrant innate immune responses critically shape self-destructive adaptive immune reactions. The complex interplay between the innate and adaptive immune systems represents a central mechanism underlying autoimmune pathologies, which cause tremendous suffering for millions of people, and has shed light on new therapeutic strategies.

The innate immune system involves many types of immune cells and uses an array of cell surface and intracellular germline-encoded innate sensors to respond rapidly to pathogen-or damage-associated molecular patterns (DAMP or PAMP). Upon activation, innate immune cells secret a multitude of cytokines, chemokines, and effector molecules to directly control infection, attract other leukocytes, and activate effector cells locally or systemically. Meanwhile, dendritic cells (DCs) take up, process, and present antigens to engage with cognate adaptive immune cells and direct effective adaptive immune responses.

Systemic lupus erythematosus (SLE) presents a prototypical systemic autoimmune disease with broad-spectrum autoantibodies and complicated multiorgan involvement but no defined etiology [3–6]. It is clear that numerous protein mediators, cells, and pathways participate in SLE pathogenesis. Hyperactivated or abnormally differentiated, T cells and B cells can synergistically enhance the development of a plenitude of autoantibodies to sustain systemic autoimmune responses [7,8]. In the past decade, plasmacytoid DCs (pDCs), a type of innate immune cells, have been linked intimately to SLE and other autoimmune diseases through their exclusive production of type I interferon (IFN), a cytokine that drives the development of systemic autoimmunity.

## pDCs Specialize in Innate Type I IFN Production

First observed by pathologists in the 1950s, human pDCs were named plasmacytoid T cells or plasmacytoid monocytes due to their plasma cell morphology and their expression of T cell and myeloid cell markers [9]. In the 1980s, a mysterious human blood cell type was demonstrated to be responsible for producing large amounts of type I IFN following culture with viruses, and these cells were called “natural type I IFN-producing cells.” In the late 1990s, human pDCs and mouse pDCs were positively identified [10], and since then research on these fascinating cells has taken off.

pDCs constitute only 0.2%–0.8% of human peripheral blood mononuclear cells but are responsible for producing more than 95% of type I IFN when cells are exposed to viral particles [11]. The human genome contains multiple genes that belong to the type I IFN family: 13 IFN-α subtypes, IFN-β, IFN-ω, IFN-τ, and IFN-κ. Activated pDCs broadly transcribe almost all type I IFNs (except IFN-κ) and type III IFNs (*i.e.* IFN-λ1–3), but not type II IFN (*i.e.* IFN-γ) [12,13]. Besides their ability to robustly and rapidly produce IFN, human pDCs produce proinflammatory cytokines, such as tumor necrosis factor α (TNF α) and interleukin 6 (IL-6), and secrete a list of chemokines to coordinate the attraction of various immune effectors in response to viral infection [12–15]. IFN secretion by pDCs is transient and non-repetitive [12], which reflects the general nature of innate immune responses and is consistent with the physiological role of pDCs during the early antiviral immune surveillance but contrasts with aberrant pDC activation under autoimmune conditions.

The mechanism by which pDCs induce the extraordinarily rigorous IFN response has been intensely investigated and several intriguing features have been revealed. First, human pDCs selectively and abundantly express TLR7 and TLR9, two innate endosomal sensors that specifically detect single-stranded RNA and unmethylated CpG DNA, respectively [16–18]. This property makes pDCs superbly sensitive to internalized nucleic acid agonists. Second, pDCs constitutively express high levels of interferon regulatory factor 7 (IRF7), the master mediator of IFN production, as well as the related IRF4 and IRF8 [12]. Pre-formed IRF7 protein allows a rapid IFN response that by passes IFNα/β receptor–mediated feedback signaling [19]. Third, pDCs are equipped with a prominent rough endoplasmic reticulum network and a unique membrane trafficking pathway, which enable effective intracellular TLR7 and TLR9 translocation, processing, and compartmentalized signaling [20–29]. As a result of these intrinsic cellular properties, pDCs readily and rigorously respond to TLR7 and TLR9 ligands.

In addition to TLR7 and TLR9, many other nucleic acid sensors have been identified in recent years, which are reviewed elsewhere [30–32]. Nucleic acids constitute a class of DAMP that is particularly important in inducing IFN. It is also worthwhile to emphasize that pDCs do not innately respond to natural DNA, RNA, or materials released from dead cells. Spontaneous IFN production by pDCs is prevented by strategic intracellular location of TLR7/9 and the ubiquitous presence of nucleases in the extracellular environment. However, this protective mechanism against innate immune response to self-nucleic acids is frequently breached in SLE patients.

## pDCs and IFN are Critically Implicated in SLE

Since the first reported detection of IFN in SLE patients [33], evidence of the association between IFN and systemic autoimmune disease has been overwhelming. Lupus patients express an “IFN signature” (*i.e.* a transcript of a panel of type I IFN–responsive genes) in peripheral blood that is generally associated with the severity of the disease [34–38]. Serum levels of IFNα are positively correlated with circulating anti-dsDNA autoantibodies and SLE disease activity index scores in childhood-onset SLE [39]. Interestingly, nearly 90% of pediatric SLE patients and more than 50% of adult patients displayed a peripheral IFN signature [35,39]. Moreover, half of biopsied glomeruli from SLE kidneys contain detectable IFN-inducible gene transcripts, which implies an IFN-mediated response in diseased organs [40]. Separately, researchers observed that patients with malignant or viral disease occasionally developed a lupus-like syndrome after IFNα administration, which suggests a causative relationship between IFN and lupus pathogenesis [35]. Many attempts have been made to identify the connection between IFN and pDCs. Studies focused on peripheral blood have found reduced numbers of circulating pDCs with certain phenotypic changes in SLE patients [35]. By contrast, abundant infiltrating pDCs have been observed in the skin lesions of cutaneous lupus patients, and activated pDCs are correlated with positive IFNα transcripts in both dermal lesions and non-inflamed skin [25]. Taken together, these findings suggest a dynamic participation of pDCs during disease manifestation and call for more detailed mechanistic characterization of pDC activities.

**Figure 1:**
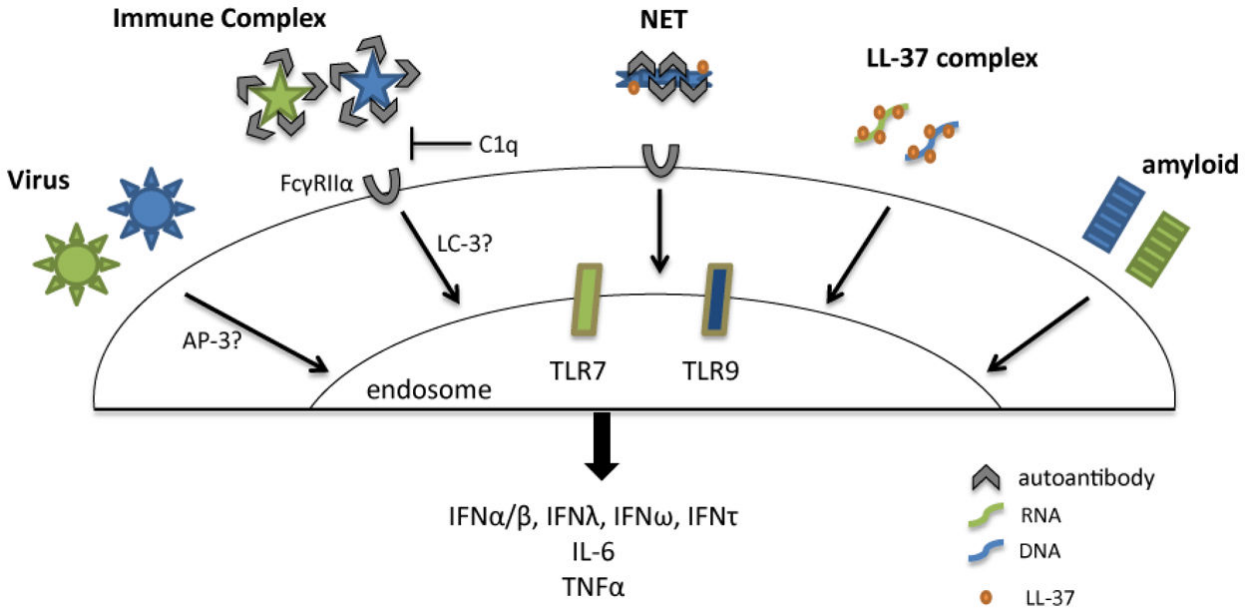
Human pDCs produce IFN in response to diverse biological stimuli.

Autoantibody production is a hallmark of various autoimmune diseases, whereas anti-dsDNA antibody is exclusively associated with SLE. Many SLE patients exhibit defects in the clearance of apoptotic cells, the debris of which can subsequently complex with autoantibodies to form circulating immune complexes (ICs) [41]. Leadbetter et al. first demonstrated that ICs containing nucleic acids can stimulate autoreactive B cells by dual engagement of B cell receptors and intracellular TLRs, thus promoting and sustaining lupus pathogenesis [42]. Although pDCs usually do not respond to dead cells, lupus ICs can be endocytosed into pDCs via binding to the Fc receptor FcγRIIα (*i.e.* CD32) and subsequently activate TLRs to induce IFN production (Figure 1). Specifically, internalized ICs containing nucleosomes engage with TLR9, whereas TLR7 in pDCs is stimulated by RNA-containing ICs made of autoantibody bound to U1 small nuclear RNA [25,35]. Deficiency of the complement component C1q represents the strongest known susceptibility factor for SLE and directly contributes to the clearance defect displayed by patients [43]. C1q-bound ICs are normally removed by myeloid cells in healthy individuals, which prevents ICs from activating pDCs [44,45]. This mechanism is therefore compromised in lupus patients with low C1q levels (Figure 1).

Autophagy is a degradation pathway that involves the engulfment of cytoplasmic contents and their delivery for lysosomal degradation. A recent study suggested that, in murine pDCs, DNA-containing ICs are transported by a process called microtubule-associated protein 1A/1 B-LC3–associated phagocytosis primarily via the convergence of phagocytic and autophagic pathways to induce IFN production [46]. This pathway unexpectedly diverges from the membrane trafficking pathway involving AP-3, which is critical for TLR9 signaling induced by viruses and synthetic nucleic acid agonists [24,26] (Figure 1). Whether a comparable signaling compartment is preserved in human pDCs and thus can be specifically targeted remains to be seen. On the other hand, the activation of pDCs through TLR7/9 by ICs stimulates the nuclear factor κB (NF-κB) pathway essential for pDC survival, which undermines the effectiveness of high-dose steroid treatment owning to the resistance by lupus pDCs [47]. By contrast, direct blockade of TLR signaling with specific TLR7/9 inhibitors provides an alternative and effective therapeutic option [47,48].

In parallel with the IFN signature, SLE blood selectively expresses a panel of genes involved in granulopoiesis that correlates with the abnormal presence of a large number of immature neutrophils [34,49]. Neutrophils are granulocytes that participate in the immediate early inflammatory response to infection or injury. Activated neutrophils can undergo a peculiar form of cell death involving neutrophil extracellular traps (NET), called NETosis, in which decondensed chromatin containing cytoplasmic proteins is expelled from the cells to form NET [50]. Intriguingly, lupus autoantibodies against ribonucleoprotein complex or antimicrobial peptide LL-37 can strongly stimulate IFN-primed neutrophils to induce NETosis [51,52]. In conjunction with anti-nuclear autoantibodies, nucleic acid–rich NET subsequently activates pDCs to trigger the production of type I IFN (Figure 1). This pDC-IFN-autoantibody-neutrophil-NET loop is self-perpetuating and likely has a critical function in sustaining aberrant IFN production and pathogenic development in SLE [53,54].

IFN is a pluripotent cytokine that has a broad effect on all nucleated cells for antiviral protection and beyond [55,56]. Blanco et al. first discovered that SLE serum can potently differentiate human monocytes into DCs that function as antigen-presenting cells to activate CD4 T cells in a type I IFN-dependent manner [57]. IFN secreted by pDCs can directly sensitize naïve B cells by upregulating TLR7 expression [58]. Activated pDCs also express CD70, which engages with its receptor CD27 on native and memory B cells to promote B cell proliferation and differentiation [59]. Furthermore, IFN and IL-6 produced by TLR-activated pDCs cooperatively induce human plasma cell differentiation [60]. Given its profound effects on T cells, DCs, neutrophils, and many other cell types, type I IFN is a central molecular mediator that propels the pathogenesis of SLE. This notion is further supported by observations that genes encoding products directly involved in TLR and IFN signaling pathways constitute a majority of genetic risk factors for SLE and that increased gene dosage of TLR7 directly results in lupus manifestation *in vivo* [3,5,61–63].

## pDCs and IFN are Involved in Other Autoimmune Pathologies

A group of diverse inheritable diseases were recently linked by their upregulated type I IFN expression and shared autoimmune features [64]. Aicardi-Goutieres syndrome is a severe inflammatory disorder that affects the brain and skin and has marked IFN production and occasionally overlapping features with SLE. Mutations in the protein three prime repair exonuclease 1 (TREX1), a major DNA exonuclease important in clearing endogenous DNA and antiretroviral infection, are responsible for Aicardi-Goutieres syndrome and familial chilblain lupus [65]. Remarkably, heterozygous mutations in TREX1 represent the single most common cause of monogenic lupus and mice lacking *Trex1* develop a severe multiorgan autoimmune disease that is driven by IFN from non-hematopoietic cells [66,67]. Spondyloenchondrodysplasia manifests with an autoimmune spectrum overlapping with SLE. Interestingly, tartrate-resistant acid phosphatase mutation associated with spondyloenchondrodysplasia increases the amount of phospholatedosteopontin, which likely augments IFN production by pDCs and leads to the development of SLE and lupus-related autoimmunity [68,69].

A comprehensive survey has identified a gene set of 36 IFN-inducible transcripts that are commonly upregulated in patients with SLE, rheumatoid arthritis, myositis, and systemic sclerosis (SSc), which suggests the involvement of IFN in a wide range of autoimmune conditions [70]. In primary Sjögren syndrome, the salivary and lacrimal glands are the targets of destructive autoimmune reactions. The increased expression of IFN-inducible genes in salivary glands correlates with the presence of infiltrating pDCs [71]. Immune thrombocytopenia is an autoimmune disorder of childhood characterized by immune-mediated destruction of platelets. A higher number of circulating pDCs is associated with the upregulated expression of IFN-inducible genes by the monocytes of patients with immune thrombocytopenia [72]. SSc is a complex disease with features of extensive fibrosis and circulating autoantibodies against various cellular antigens. SSc patients display a detectable IFN signature and serum IFNα, which are associated with the vascular pathology and fibrotic process [73]. Transgenic mice expressing a mutant *FBN1* gene, which encodes a mutated form of fibrillin-1 responsible for stiff skin syndrome, exhibit features of SSc with aggressive skin fibrosis [74]. In these animals, IFN-producing pDCs have been found to infiltrate the affected skin together with CD4 T cells and plasma cells, which presumably promote autoantibody production [74].

pDCs acutely infiltrate in response to skin injury and assist the wound-healing process under normal conditions [75]. In addition to the diseases described above, IFN-producing pDCs are common also in other cutaneous autoimmune diseases, in which cytotoxic attack leads to degeneration of the basal epidermal layer [76]. For psoriasis, a cutaneous autoimmune inflammatory condition, an IFN signature was detected in the psoriatic plaques and IFN could facilitate the spread of the lesions [25]. The group lead by M. Gilliet demonstrated that pDC-derived IFN is essential for the development of psoriasis [77]; moreover, antimicrobial peptide LL-37 within the psoriatic skin can complex with self-nucleic acids and deliver them to pDCs to engage TLR7/9 and induce IFN production [78] (Figure 1). Separately, significant upregulation of the type I IFN pathway in blood cells is associated with dermatomyositis, a severe autoimmune disease involving muscle, skin and vasculature [79]. Increased number of pDCs and amounts MxA protein are present in both skin and muscle from patients with juvenile dermatomyositis [80]. Collectively, these studies highlight the prominent involvement of IFN-secreting pDCs in a variety of autoimmune diseases.

## Physiological Functions of pDCs – *In vivo* Studies

*In vivo* functional studies of pDCs have been hindered by technical limitations. pDCs are rare bone marrow–derived leukocytes (<1% in any tissue or organ) that express markers that overlap with cells of other lineages (*e.g.* B220, CD11c, Gr-1). Early attempts to deplete pDCs with anti-Gr-1 antibody resulted in nonspecific ablation of many cells, including granulocytes. Mouse pDCs abundantly express BST-2 and Siglec-H, two markers useful for positive detection of pDCs in a naïve host. However, BST-2 can be induced in most cell types following stimulation with IFNα/β or IFN-γ [81]. As a result, anti-BST-2 antibodies (including 120G8 and mouse plasmacytoid dendritic cell antigen-1 (mPDCA-1)), may also deplete additional cell types when injected during an infection or inflammatory response. Recently, several mouse lines were developed to facilitate specific genetic ablation of pDCs *in vivo*, including BDCA2/CLEC4C-DTR mice [82], conditional knockout mice carrying LoxP-flanked E2-2 allele (Itgax-Cre^+^Tcf4^flox/−^) [83,84], and Siglec-H-DTR mice [85]. Unfortunately, diphtheria toxin treatment of Siglec-H-DTR mice also ablates marginal zone macrophages and DC precursors, underlining yet again the necessity to use precaution when interpreting *in vivo* experiments [86]. Another mouse strain, *feeble*, carries a point mutation in peptide/histidine transporter solute carrier family 15, member 4 (SLC15A4) [24]. *Feeble* mice display selective deficiency in IFN production by pDCs and thus are valuable in examining the function of the pDC-IFN pathway under different conditions *in vivo*.

Consistent with initial findings from human studies, murine pDCs are involved in protecting against infection from an array of viruses *in vivo*; this protection is associated with the cells’ IFN production in local tissues or systemically [87]. A detailed characterization of BDCA2/CLEC4C-DTR mice indicated that pDCs are especially important in eliciting early IFN production in response to murine cytomegalovirus, vesicular stomatitis virus and herpes simplex virus, which restrict viral replication and enhance the function of natural killer cells and virus-specific cytotoxic T lymphocytes [82,88]. In addition, conditional knockout mice constitutively depleted of pDCs in peripheral lymphoid organs and tissues exhibited a severely defective ability to control acute infection with the cytopathic mouse hepatitis virus [84]. Persistent viral infection poses a continuous challenge to human health. Two studies illustrated that pDCs, likely via their secreted type I IFN, are crucial to mediate the effective T cell responses to resolve persistent lymphocytic choriomeningitis virus (LCMV) infection [84,89].

A number of studies have revealed a tolerogenic function of pDCs *in vivo*. pDCs in liver tissue and mesenteric lymph nodes play a non-redundant role in initiating oral tolerance to proteins or molecules absorbed by the gut [90]. Depletion of lung pDCs leads to the development of asthmatic reactions to inhaled inert antigens [91]. Moreover, pDCs effectively present alloantigens associated with vascularized grafts to mediate tolerance to transplantation [92]. One mechanism responsible for tolerance induction is the promotion by pDCs of regulatory T cell generation after presentation MHC-II restricted antigens [93–95]. In addition, a population of pDC-like CD11c^+^B220^+^CD19^+^cells in tumor-draining lymph nodes were found to secrete indoleamine 2,3-dioxygenase, a tryptophan metabolic byproduct with potent immune suppressive ability [96].

In spite of the regulatory function, pDCs have been shown to directly participate in organ-specific autoimmune responses. In draining pancreatic lymph nodes of young non-obese diabetic (NOD) mice, a prominent IFN signature is correlated with the presence of IFNα-secreting pDCs [97]. Blockade of IFNα/β receptor significantly delays the onset and incidence of autoimmune type I diabetes (T1D), suggesting an essential role of the pDC-IFN pathway in initiating T1D in the NOD model. Moreover, IFN-α-producing pDCs co-infiltrate and directly interact with B-1a cells and neutrophils in the pancreas of young NOD mice; this cross-talk is essential to the initiation of the diabetogenic T cell response and T1D development [98]. Interestingly, an integrated genome-wide analysis of human T1D revealed an IRF7-driven inflammatory network mimicking an antiviral response that may contribute to the risk of T1D [99]. Whether human pDCs are involved in such inflammation remains to be shown. However, in the T cell–dependent experimental encephalomyelitis autoimmune mouse model, conflicting observations have been reported regarding the functional involvement of pDCs [93,100–102].

## Role of pDCs in Mediating Pathogenesis of Experimental Lupus

### Spontaneous lupus models

Animal models are valuable tools for dissecting the dynamic and kinetic interactions between immune cells at different stages of disease progression and for characterizing the spatial and functional involvement of cells or molecules in unique tissue and organ environments. The most commonly studied experimental lupus models are inbred mouse strains that spontaneously develop a disease that shares characteristics with human SLE [103–107]. Numerous studies have demonstrated clearly the defects in B cell and T cell compartments that sustain autoreactivity and disease manifestation. The roles played by IFN and pDCs have been scrutinized intensely in the past decade and intriguing insights have been obtained.

**Figure 2:**
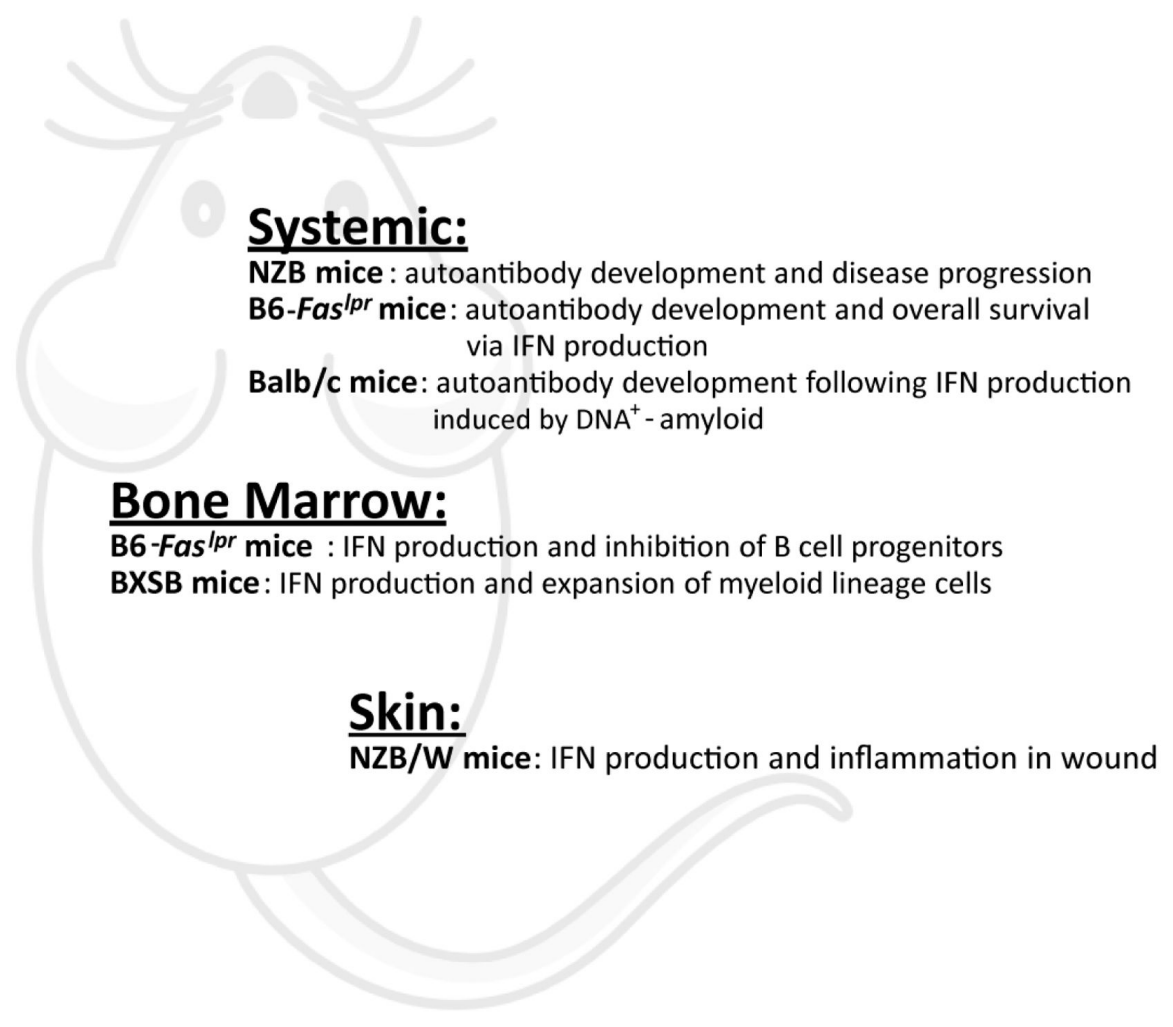
pDCs critically promote the pathogenesis of murine lupus.

NZB/W F1 mice, a well studied lupus-prone strain, harbor multigenic mutations that predispose them to autoimmune development. Systemically induced IFNα/β production in young NZB/W F1 mice as well as B6.Sle123 mice significantly accelerates lupus nephritis [108–110]. Although NZB/W F1 mice lack the prominent IFN signature associated with clinical lupus, IFNα/β receptor deficiency or TLR7/9 inhibition greatly ameliorate lupus, suggesting the importance of nucleic acid sensing in promoting pathogenic development [48,111]. A recent unexpected finding was that IFNβ is dispensable for autoantibody production and disease progression in NZB/W F1 mice [112], implying a functional redundancy within type I IFN family and the importance of IFNα subtypes in this model. More recently, Baccala et al. reported that IRF8-deficient NZB mice lacking pDCs failed to develop anti-nuclear, anti-chromatin, and anti-erythrocyte autoantibodies and had limited kidney disease [113] (Figure 2). pDC deficiency not only reduces the number of T cells but also selectively diminishes the accumulation of CD21^−^CD23^−^ extrafollicular B cells in the spleen. Age-associated B cells, which lack CD21 and CD23 but express additional myeloid cell-specific markers, have been detected in many lupus-prone mouse strains and presumably develop in response to TLR7 activation [114–116]. These results highlight a pivotal role of the pDC-IFN pathway in the development of systemic autoimmunity *in vivo* and suggest a significant effect of pDCs on the humoral autoimmune response. In addition to systemic immune reactions, pDCs and their secreted type I IFN after TLR7/9 activation play a key role in mediating the prolonged inflammation and chronic lesions on tape-stripped skin of NZB/W F1 mice, a condition that mimics cutaneous lupus [117] (Figure 2). The involvement of pDCs in this pathological process is distinct from their transient IFN response during wound healing in normal mice [75,117]. Therefore, pDCs apparently participate in different aspects of autoimmune development in the NZB/W F1 model.

Mice homozygous for the lymphoproliferation mutation (*Fas*^lpr^) spontaneously develop systemic autoimmunity, lymphadenopathy associated with proliferation of aberrant T cells, and IC glomerulonephrosis. Unlike its attenuating effect on NZB/W F1 mice, IFNα/β receptor–deficiency endorses MRL-*Fas*^lpr^ mice to develop exacerbated lymphoproliferation, autoantibody production, and end organ disease [118]. Instead, IFNγ has a predominant role in autoimmune-associated disease development in these animals [118–121]. To understand the overt contribution by DCs, Teichmann et al. analyzed DC-deficient MRL-*Fas*^lpr^ mice (controlled by CD11c:DTA), in which >90% of classical DCs and >80% of pDCs in spleen were ablated, and reported that, while required for T cell expansion and differentiation, DCs are critical to maintain the high levels of autoantibodies and number of short-lived antibody-secreting plasmablasts [122]. The same research group reported an additional function for MyD88-stimulated DCs in promoting dermatitis and renal inflammation [123]. Interestingly, pDCs in the bone marrow of adult MRL-*Fas*^lpr^ mice express high levels of IFNα mRNA in a MyD88-dependent manner, which possibly contributes to the inhibition of B cell progenitor cells [123] (Figure 2). Despite the reported phenotype of IFNAR^−/−^ MRL-*Fas*^lpr^ mice, prophylactic administration of anti-IFNα/β receptor blocking antibody in young MRL-*Fas*^lpr^ mice provides transient protection against the escalation of anti-RNP autoantibody titers and proteinuria, suggesting a possible role of type I IFN in promoting lupus development at the initiation phase [112]. More recently, Baccala et al. obtained pDC-defective C57BL/6-*Fas*^lpr^ mice, in which *feeble/*Slc15a4 mutation selectively disrupts pDC-mediated IFN response, and observed the disappearance of autoantibodies, reduced lymphadenopathy and splenomegaly, decreased numbers of T cells and age-associated B cells, and prolonged survival when comparing with C57BL/6-*Fas*^lpr^ mice [113] (Figure 2). Altogether, the pDC-IFN pathway plays important and complex roles in the *Fas*^lpr^-mediated lupus model. Further characterization is necessary to fully reveal the functional involvement of this pathway at specific stages of disease development.

BXSB inbred male mice harbor the Y-linked autoimmune accelerator locus (*Yaa*) with duplicated chromosome segment containing TLR7, which is primarily responsible for the autoimmune phenotype [124–126]. Analysis of mice overexpressing TLR7 revealed constitutive expression of type I IFN mRNA by pDCs in bone marrow, which presumably drives the proliferation of Sca-1^+^ granulocyte/macrophage progenitors and subsequent expansion of peripheral myeloid cells [127] (Figure 2). However, it is unclear if pDCs in the peripheral tissues also intrinsically produce IFNα/β in these mice thus contribute to lupus manifestation. Nevertheless, therapeutic treatment of BXSB mice with an anti-type I interferon receptor antibody diminished their autoimmune disease, suggesting a functional involvement of IFN signaling in autoimmune pathogenesis of this model [112].

### Inducible lupus models

SLE is rarely a single-gene disorder. Although genetic risk factors clearly contribute to lupus, a significant portion of patients do not display any known mutations in their genomes [128]. On the other hand, stochastic stimuli and environmental factors (*e.g.* infections, chemical compounds, somatic mutations, drugs, and aging [129–133]) play roles that exacerbate autoimmune susceptibility and, at times, stimulate immune responses that lead to systemic autoimmunity. An immunocompetent host employs a multitude of regulatory mechanisms (*e.g.* negative selection, anergy, receptor editing, and suppressor cells) to minimize autoreactivity that would cause damage and harm to itself. Understanding the mechanism by which immune tolerance is breached by an exogenous trigger is crucial for identifying the essential pathways responsible for the establishment of autoimmunity.

It is well known that significant fraction of newly generated and mature B cells shows some degree of autoreactivity [134,135]. Even so, humoral immune tolerance is largely maintained even after challenges with autoantigens. In one study, immunization of bacterial DNA in the presence of a carrier protein in non-autoimmune mice induced the production of anti-bacterial DNA antibody lacking reactivity to mammalian DNA [136]. In another study, wild-type mice that received an injection of a large number of apoptotic human cells developed modest and transient autoantibody production without exhibiting clinical changes [137]. HMGB1-nucleosome complexes constitute the major nuclear component recognized by SLE autoantibodies. However, after inoculation into non-autoimmune mice, these complexes induced a limited antibody response against the immunized components without overt lupus-like disease [138].

The innate immune response instructs the corresponding adaptive immune response. For effective antibody induction, various adjuvants with strong innate stimulatory activities have been used to boost the B cell response and generate high titers of immunogen-specific antibodies in experimental settings and effective vaccines. The role played by innate immune activation in initiating the autoreactive humoral response has not been extensively studied. Hydrocarbon oil pristine triggers profound inflammation and IFN production; the latter is essential to the development of autoantibodies and glomerulonephritis in non-autoimmune mice [139,140]. This finding thus highlights an equally critical role of type I IFN in the induced and spontaneous systemic autoimmunity.

Amyloid is formed from a native protein after a process of aberrant aggregation and misfolding [141,142]. Amyloid fibrils contain extensive β sheet structures and can be found extracellularly or intracellularly. Amyloid depositions *in vivo* are often heterogeneous and contain non-proteinaceous cofactors [141,143], which may be explained by the fact that amyloid precursor proteins display an intrinsic affinity towards nucleic acids and glycosaminoglycans, an interaction that promotes the rapid formation of amyloid [144]. We examined the innate immune properties of amyloid fibrils containing nucleic acids and found that these complexes are potent inducers of type I IFN from human pDCs [145] (Figure 1). Regardless of their source or type, nucleic acids incorporated into amyloid fibrils could be efficiently internalized by human pDCs into an endosomal compartment to trigger TLR activation and strongly induce IFN production. Because amyloid proteins use a variety of mechanisms to penetrate cells, amyloid fibrils containing nucleic acids trigger IFN production independently of FcγRIIα [145].

When inoculated into the peritoneal cavity, DNA-containing amyloid fibrils induced selective pDC infiltration, which was associated with a predominant type I IFN response. After immunization with DNA-containing amyloid fibrils, non-autoimmune mice developed stable anti-nuclear autoantibodies and abroad autoreactive humoral response against DNA, RNA, Sm/RNP, and histone [145]. Proteinuria and antibody depositions in the glomeruli of the kidneys were also detected, suggesting the development of a lupus-like syndrome. In amyloid-immunized mice, the establishment of anti-nuclear serology requires the signaling of IFNα/β receptors. We found that pDCs were indispensable for not only the acute IFN response but also the subsequent autoantibody development [145]. By contrast, pDCs were not involved in the induction of antibodies against the amyloid protein *per se*, and pDC depletion did not affect the development of proteinuria. Therefore, IFN-producing pDCs play an essential and selective role in instigating the humoral autoimmune response following a strong innate immune activation (Figure 2).

Whereas pDCs and IFN appears to influence B cell differentiation *in vitro* (see earlier section), our data suggests that pDCs are largely dispensable for the immunogen-specific IgG response *in vivo*. Consistent with this observation, Baccala et al. reported that *feeble* mice elicited normal T cell–dependent and –independent IgG responses that were indistinguishable from those of wild-type mice [113]. In addition, it has been shown that neither pDCs or IFN was required to elicit a protective antibody response after inoculation with live attenuated flu vaccine [146]. By contrast, pDCs and IFN were critical for the generation of primary IgG and IgA response after immunization of inactivated whole virus flu vaccine [146]. On the other hand, pDCs are suggested to have a role in IgA production under steady-state conditions, based on the observation that, when co-cultured with mucosal B cells, pDCs from mesenteric lymph nodes and Peyer’s patches can facilitate T cell–independent IgA secretion via production of APRIL and BAFF [147]. Remarkably, during intestinal rotavirus infection, pDC-derived type I IFN was required for optimal B cell activation and virus-specific IgA antibody secretion for effective protection [148]. Therefore, pDCs seem to have multifarious effects on humoral responses. Further investigation on how pDC-IFN pathway selectively instructs autoreactive B cell selection and expansion would shed light on the key processes involved in the breakdown of immune tolerance.

The fact that nucleic acid-containing ICs and autoantibody-induced NET potently trigger IFN production from pDCs implies a role of type I IFN at late stage of lupus pathogenesis after the establishment of humoral autoimmunity. However, our results demonstrate that IFN also function as a critical mediator in the early stage of autoimmune development, a conclusion further supported by the finding that IFNα/β receptor blockade was effective in BXSB mice only when administrated to young mice at a preclinical phase [112]. These studies indeed suggest a therapeutic window of IFN blockade, which may be more effective at the onset of autoimmune response.

For a long time, the presence of amyloid was exclusively associated with about two dozen human pathologies with Alzheimer’s disease as the best known example [149,150]. However, an increasing number of so-called functional amyloids have been shown to participate in diverse normal cellular functions, suggesting a prevalence of this peculiar form of protein post-translational modification [151–158]. Amyloid fibrils also represent a type of DAMP that is capable of activating inflammasome thus contributes to autoinflammatory responses [159]. Therefore, it would be important to examine the direct involvement of amyloid in disease pathogenesis among SLE patients.

## Conclusion

As exemplified by SLE, systemic autoimmune diseases present a supreme challenge for immunologists because the pathogenic processes involve not only different cell types, numerous molecular mediators, and multiple organs but also are controlled by discrete mechanisms at various stages of development. The discoveries of predominant involvement of type I IFN and nucleic acid sensing mechanisms have driven the development of therapies targeting IFNα/β or TLRs, many of which are currently under clinical evaluation [160,161]. Needless to say, the success of new therapies relies on a deep understanding of the participation of specific cellular and molecular pathways in the disease progression.

From earlier human studies, pDCs were regarded as the principal IFN producer in SLE and only recently were revealed as an essential component in promoting lupus development at multiple stages and in different tissues. In the upcoming years, we anticipate to see further elucidation of the functions of pDCs in stimulating autoreactive B cell development, mediating tissue-specific inflammation or damage, and sustaining ongoing autoimmunity. Because pDCs readily interact with many types of cells, functional cross-talk between pDCs and neutrophils, B cells, or T cells during lupus pathogenesis *in vivo* should be investigated in details. The effort may reveal novel molecular and therapeutic targets useful for blocking the development of autoimmune diseases.

Besides LL-37 and amyloid precursor proteins, we do not know whether there exist other endogenous carriers that are capable of enabling nucleic acid internalization and abnormal innate activation of pDCs. Searching for these molecules by *in vitro* screening and identifying them directly from patients would significantly expand our understanding of the factors that drive the initiation and progression of the autoimmune response. Being a complex disease, SLE likely represents a convergence of autoimmune disorders with diverse etiological causes. Understanding the nature of initial triggers that provoke aberrant innate immune responses will help eventually in developing effective personalized therapeutic strategies in the future.
